# Comparative analysis of the aberrant immunophenotype and clinical characteristics in dogs with lymphoma: a study of 27 cases

**DOI:** 10.3389/fvets.2023.1254458

**Published:** 2023-10-16

**Authors:** Hyeona Bae, Sang-Ki Kim, DoHyeon Yu

**Affiliations:** ^1^College of Veterinary Medicine, Gyeongsang National University, Jinju, Republic of Korea; ^2^College of Industrial Science, Kongju National University, Yesan, Republic of Korea

**Keywords:** lymphoma, immunophenotyping, flow cytometry, aberrancies, dog

## Abstract

**Introduction:**

Aberrant lymphoma phenotypes are frequently found in dogs, but the clinical implications are sparse.

**Methods:**

Twenty-seven dogs with aberrant lymphoma diagnosed using flow cytometry between 2017 and 2023 were analyzed. Major paraneoplastic syndromes, prognostic factors, and clinical features of lymphoma were compared to their immunophenotypes.

**Results:**

Twenty-seven dogs had aberrant immunophenotypes, with MHCII- (48%) and CD3+/CD21+ (44%) being the most commonly identified aberrancies. In B-cell lymphoma, the most frequent aberrancies were MHC II- (53%), CD3+/CD21+ (41%), CD34+ (24%), and CD79a- (24%). Meanwhile, in T-cell lymphoma, CD3+/CD21+ (63%), CD4-/CD8-(50%), CD5- (50%), and CD45- (50%) were the most common. The platelet–neutrophil ratio was significantly higher in the CD3+/CD21+ group than in the other groups, where either one or both markers were not expressed (55.23 ± 39.64; 18.72 ± 14.95, respectively; *p* = 0.001). Serum albumin concentration was significantly lower in the MHCII-group (2.59 g/dL, 95% CI 2.31–2.87) than in the MHCII+ group (3.06 g/dL, 95% CI 2.88–3.23; *p* = 0.009). CD34 expression showed significant correlations with cranial mediastinal mass, WHO clinical substage, and fever (*p* = 0.028, *p* = 0.041, and *p* = 0.047, respectively). MHCII expression was correlated with adverse reactions to chemotherapy, cranial mediastinal masses, and fever (*p* = 0.009, *p* = 0.023, and *p* < 0.001, respectively). No statistically significant differences in the survival period were observed for any of the phenotypic aberrancies.

**Conclusion:**

Aberrant lymphomas are common in dogs. Some clinical prognostic factors that significantly correlate with aberrant immunophenotypes have been identified and can be applied clinically.

## 1. Introduction

When compared to experimental animals, naturally occurring diseases in dogs could reflect human diseases such as cancer. Studying these similarities can provide valuable insights into disease mechanisms, treatments, and potential therapeutic targets for dogs as well as humans.

Immunophenotyping plays a crucial role in the accurate diagnosis and classification of canine lymphoma, similar to human lymphoma. Despite of limited availability of commercially specific dog antibodies, a significant prognostic factor based on the immunophenotyping in canine lymphoma has been established (1–3). The immunophenotypes of lymphoma in dogs is categorized according to the origin of B-and T-cells, with representative markers of CD21/CD79a and CD3/CD4/CD8 commonly used in dogs. Furthermore, various phenotypes have been identified in detail, such as CD45 in all leukocytes, CD34 in precursor hematopoietic cells, and major histocompatibility complex class II (MHCII) in antigen-presenting cells. Among these, aberrant phenotypes characterized by either increased or decreased expression of specific antigens are well established in both human and veterinary medicine (4–7). Several studies are being conducted to explore the possibility of utilizing immunophenotypes, including various aberrancies, for clinical purposes and prognosis prediction in humans (8–13).

Clinical prognostic factors such as World Health Organization (WHO) substage, mediastinal lymphadenopathy (14), and paraneoplastic syndrome (PNS) (15) have been studied in canine lymphoma, but the clinical implications of aberrant phenotypes have yet to be studied in dogs.

Thus, the aims of this study were [1] the identification of aberrant phenotypes in dogs with various types of lymphoma and [2] the investigation of associated types with the severity of clinical signs, PNS, and prognosis of the aberrant phenotypes.

## 2. Materials and methods

A retrospective *in vitro* analysis of the clinicopathological parameters and immunophenotypes of dogs with lymphoma was conducted using lymph node aspirates and peripheral whole blood samples collected at the time of diagnosis. This study was approved by the Institutional Animal Care and Committee (IACUC) GNU-230425-D0087.

Among the dogs diagnosed with lymphoma that visited Gyeongsang National University Veterinary Teaching Hospital between 2017 and 2023, 35 dogs that were immunophenotyped by flow cytometry were included in this study. The inclusion criteria were as follows: [1] dogs diagnosed with lymphoma through the following diagnostic procedures: cytology, histopathology, immunophenotyping, and clonality test (through fine needle aspiration (FNA) or a biopsy sample of enlarged lymph nodes or target lesions); [2] dogs without underlying diseases other than lymphoma that may affect hematological changes; and [3] dogs with naïve lymphoma who had not received chemotherapy prior to admission or dogs with relapsed lymphoma six months after the last chemotherapy. Both nodal and extranodal forms were included, and all dogs were staged according to the WHO staging system (16). Cytologic grading was evaluated according to the updated Kiel classification (17, 18). Histopathology and polymerase chain reaction for antigen receptor rearrangement were requested from an external laboratory (IDEXX, Westbrook, ME, United States), while cytology and immunophenotyping using flow cytometry.

The classification criteria for each lymphocyte lineage in immunophenotyping were as follows: [1] B-cell lymphoma, if the tumor cells were CD21+ (>70% of the major cells) and the T-cell marker was negative; [2] T-cell lymphoma, if the tumor cells were CD3+ (>70% of the major cells) and the B-cell marker was negative; and [3] non-B and non-T lymphomas, if the tumor cells were negative for both B-cell and T-cell markers.

Phenotypic aberrancies were defined as follows: [1] reduced or absent expression of pan-leukocyte or lymphocyte markers (CD45 and MHCII), [2] simultaneous expression of lymphocyte markers of different lineages (CD3 and CD21) or different stages of differentiation (markers of mature stage and CD34), [3] in T-cell, CD4 and CD8 markers were expressed simultaneously, or neither was expressed, [4] in B-cell, loss of CD79a, which is expressed in all maturation stages of B-cells (7, 19, 20).

At the time of diagnosis, the presence of PNS was assessed in all dogs to determine the clinical course and prognosis. The correlation between WHO substage ‘b’ (clinically ill), anemia, hypercalcemia, thrombocytopenia, and immunophenotype was analyzed. Survival time was defined as the period from the date of diagnosis at our hospital to the day of death due to lymphoma. Dogs that died from causes unrelated to the tumor or whose follow-up was discontinued were considered ‘censored’. Treatment response was evaluated in dogs that had received chemotherapy or equivalent medication and was divided into complete remission (CR), partial remission (PR), progressive disease (PD), and stable disease (SD) according to previous literature (21).

Upon admission, laboratory examinations were performed to assess the overall clinical condition of the dogs and to detect any underlying diseases, including PNS. A complete blood count (Procyte Dx hematology analyzer, IDEXX, Westbrook, ME, United States) and blood film analysis, including platelet-to-lymphocyte ratio (PLR), platelet-to-neutrophil ratio (PNR), lymphocyte-to-monocyte ratio (LMR), and neutrophil-to-lymphocyte ratio (NLR) were performed. Acid–base balance and electrolyte concentrations (Nova pHOX analyzer, Nova Biochemical, Waltham, MA, United States), serum biochemical analysis (Catalyst Dx® Chemistry Analyzer, IDEXX, Westbrook, ME, United States), coagulation tests (Coag Dx™ analyzer with citrate PT and citrate aPTT cartridges, IDEXX), and complete urinalysis (VetLab UA Analyzer, IDEXX) were also performed. The fibrinogen levels were evaluated using the Millar’s method (22).

FNA aspirates were collected from the prescapular or inguinal lymph nodes of dogs with generalized lymphadenopathy, and peripheral blood was collected to evaluate WHO staging. For cases of extranodal lymphoma, FNA aspirates were collected from regional lymph nodes suspected of involvement near the target lesions (stomach, intestinal segments, liver, spleen, and cutaneous lesions) and pleural or abdominal free fluids were also collected. All aspirates were suspended in 0.3–0.5 mL of 5% dextrose saline, and peripheral blood was collected in ethylene-diamine-tetraacetic acid tubes. All samples were analyzed within 24 h of collection by the same operator, and those with ambiguous diagnoses, low cellularity, or low viability were excluded.

The antibodies used in this study were based on previous studies (23). Multi-color flow cytometric analysis was conducted to evaluate the contemporary expression of different antigens within the same cellular population. The sample preparation and analysis method was similar to previous studies (6), with the exception that CD14-negative cells were sorted (CD14 is expressed on monocytes and macrophages), and only lymphocytes were selected using the difference in granularity and lymphocyte-specific markers. The major cells were first identified through cytology, and flow cytometric analysis was primarily performed on lymphocytes, which constituted the largest population (>60%). Lymphocytes exhibiting a tumorigenic phenotype, even at a small percentage, were also analyzed. The samples were acquired using the MACSQuant Analyzer 10 (Miltenyi Biotech, Bergisch Gladbach, Germany) and analyzed using FlowJo software version 10.8.0 (Ashland, OR, United States).

All statistical analyses (Student’s t-test, Mann–Whitney U test, Fisher’s exact test, and Kaplan–Meier curve) were performed using SPSS Statistics version 27.0, for Windows (SPSS Inc., Chicago, IL, United States). Clinical data on admission, including signalment, temperature, body weight, presence of abnormalities in hematological parameters, and clinical substages, were evaluated for their impact on survival time using Kaplan–Meier estimators and log-rank tests. For Fisher’s exact test, clinical, clinicopathological, and immunophenotypic data were dichotomized and evaluated. The *p* values were two-sided and were considered significant at *p* < 0.05.

## 3. Results

### 3.1. Study population and prevalence of immunophenotypic aberrancies

The characteristics of the dogs included in this study are summarized in Supplementary Table S1. Among the dogs initially recruited for the study, 27 were included, all of whom showed immunophenotypic aberrancies with immunophenotyping by flow cytometry at the time of diagnosis. The mean (± standard deviation) age of 27 dogs was 9 ± 3.5 years (range, 3–15 years). The breeds included Maltese (*n* = 7), Mixed (*n* = 5), Shih-Tzu (*n* = 4), Golden Retriever (*n* = 3), Miniature Poodle (*n* = 2), Yorkshire Terrier (*n* = 1), Dogo Canario (*n* = 1), Dachshund (*n* = 1), Chihuahua (*n* = 1), Cocker Spaniel (*n* = 1), and Shiba (*n* = 1). The multicentric lymphoma was the most common (*n* = 24), followed by as an extranodal lymphoma, the alimentary (*n* = 1), tongue (*n* = 1), and liver (*n* = 1) forms were identified. When classified by WHO clinical stage, one was WHO stage I, two were stage III, 13 were stage IV, and 11 were stage V. Of the 27 dogs, 11 were WHO substage ‘a’ (asymptomatic) and 16 were substage ‘b’ (clinically ill).

A total of 21 dogs received chemotherapy after being diagnosed with lymphoma. Among these, 14 dogs underwent the L-CHOP protocol, three dogs were administered chlorambucil, and protocols CHOP, COP, and doxorubicin alone were applied to one dog each. Two of the dogs that received the L-CHOP protocol, died of tumor progression and were lost to follow-up immediately after chemotherapy in the first week (L-asparaginase and vincristine). One dog that was treated with chlorambucil showed a poor response and was treated with a combination of prednisolone, imatinib, and cyclophosphamide. Upon evaluation of the response after chemotherapy, five dogs with CR, six dogs with PR, four dogs with SD, and 8 with PD were identified.

On cytological examination, 12 dogs had large cells, 7 dogs had intermediate cells, and five dogs had small cells. In three dogs, intermediate and large cells were identified as heterogeneous. Among the 22 dogs that could be analyzed by the updated Kiel classification, five dogs (23%) were found to have low-grade lymphoma and 17 dogs (77%) were found to have high-grade lymphoma (Supplementary Tables S2, S3). In the low-grade lymphoma group, there were three clear cells, one prolymphocytic-like cell, and one centrocytic-like cell type. In the high-grade lymphoma group, there were seven centroblastic polymorphic (predominantly large cells), three Burkitt-type, three plasmacytoids, two pleomorphic, one centroblastic polymorphic (predominantly small cells) cell, and one anaplastic lymphoma. Of the eight dogs that could be diagnosed through histopathological and immunohistochemical methods, diffuse large B-cell lymphoma (DLBCL) was identified in five, TZL in two, and diffuse small B-cell lymphoma in one.

A total of 27 dogs were identified as having an aberrant immunophenotype (Table 1). Among the total aberrancies, MHCII- (13/27, 48%) and CD3+/CD21+ (12/27, 44%) were the most frequently identified, whereas 19% of these dogs (5/27) did not express CD3 or CD21. All 22 dogs that could be analyzed by the updated Kiel classification showed immunophenotypic aberrancies. Among the dogs that showed aberrancy, five were found have low-grade lymphoma, and 17 were found to have high-grade lymphoma, with the high-grade lymphoma group showing more immunophenotypic aberrancies. CD3+/CD21+ (4/5, 80%) and MHCII- (7/17, 41%) were the most frequently identified in low-and high-grade lymphomas, respectively. The most represented aberrancies in B-cell lymphoma were MHCII- (9/17, 53%), CD3+/CD21+ (7/17, 41%), CD34+ (4/17, 24%), CD79a- (4/17, 24%), while CD3+/CD21+ (5/8, 63%), CD4-/CD8- (4/8, 50%), CD45- (4/8, 50%), CD5- (4/8, 50%) were expressed in T-cell lymphoma. In the two cases showing the non-B/non-T phenotype, CD3-/CD21-, CD4-/CD8-, expression of CD5, and MHCII-were confirmed at a rate of 50%. In both cases, the canine natural killer cell (NK cell) marker, NKp46, was highly expressed, suggesting NK cell-derived lymphoma.

**Table 1:** Aberrant phenotype epidemiology.

Aberrancy	Percentage (aberrant/tested) (%)			
	Total (*n* = 27/35)	B-cell (*n* = 17/23)	T-cell (*n* = 8/10)	Non-B & non-T-cell (*n* = 2/2)
CD3+/CD21+	12/27 (44%)	7/17 (41%)	5/8 (63%)	0/2
CD3-/CD21-	2/27 (7%)	1/17 (6%)	0/8	1/2 (50%)
CD4+/CD8+	1/27 (4%)	0/17	1/8 (13%)	0/2
CD4-/CD8-	5/27 (19%)	–	4/8 (50%)	1/2 (50%)
CD5+	–	0/17	–	1/2 (50%)
CD5-	–	–	4/8 (50%)	1/2 (50%)
CD79a-	-	4/17 (24%)	–	0/2
CD45-	4/27 (15%)	0/17	4/8 (50%)	0/2
CD34+	5/27 (19%)	4/17 (24%)	1/8 (13%)	0/2
MHCII-	13/27 (48%)	9/17 (53%)	3/8 (38%)	1/2 (50%)

MHCII, major histocompatibility complex class II.

### 3.2. Correlation with aberrant immunophenotype and clinicopathologic, paraneoplastic syndrome measurements

**Figure 1:**
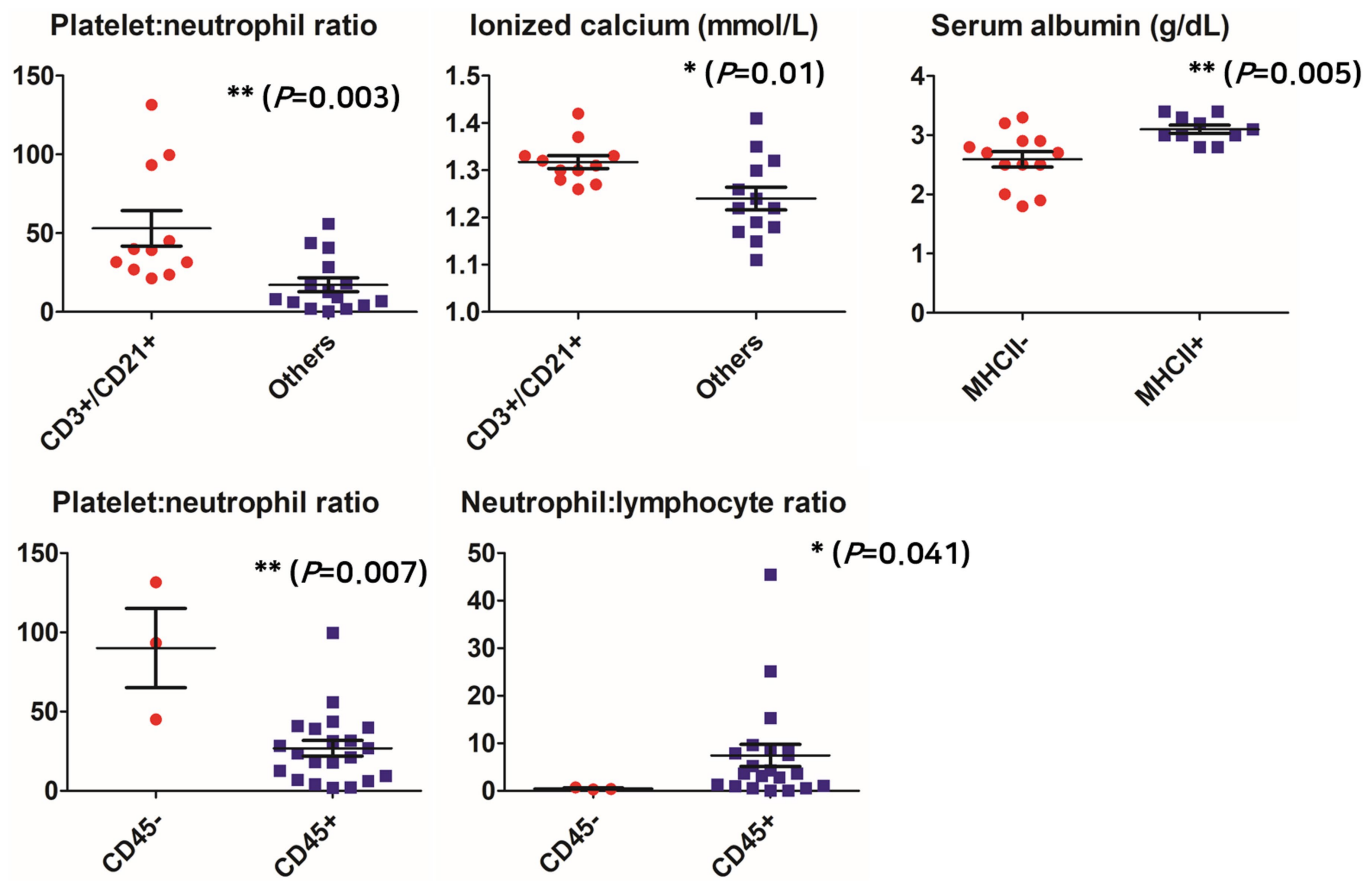
Comparison of clinicopathological parameters according to aberrant phenotypes. *p < 0.05, **p < 0.01, ***p < 0.001.

As a result of analyzing the clinical measurement in the CD3+/CD21+ group, which was identified as most frequent among the aberrant phenotypes, the PNR was significantly higher in the group identified as CD3+/CD21+ than in the other groups (either one or both were not expressed) (55.23 ± 38.52; 18.13 ± 17.12, respectively; *p* = 0.003), CD45-group than in CD45+ group (90.01 ± 43.30; 26.85 ± 22.66, respectively; *p* = 0.007). NLR was significantly lower in the CD45-group (7.45 ± 10.58; 0.47 ± 0.22; *p* = 0.041). The ionized calcium concentration was also significantly higher in the group identified as CD3+/CD21+ than in the other groups (1.32 mmol/L, 95% CI 1.29–1.35; 1.24 mmol/L, 95% CI 1.19–1.29, respectively; *p* = 0.01). Serum albumin concentration was significantly lower in the group of MHCII- (2.59 g/dL, 95% CI 2.31–2.87) than in the group of MHCII+ (3.10 g/dL, 95% CI 2.94–3.26; *p* = 0.005) (Figure 1).

When analyzing the association between phenotypic aberrancies and clinical measurements, the expression of CD34 showed significant correlations with cranial mediastinal lymphadenopathy, WHO clinical substage, and fever (*p* = 0.017, *p* = 0.037, and *p* = 0.033, respectively). Cranial mediastinal lymphadenopathy was present in four out of the five dogs (80%) that expressed CD34, whereas only three of the 18 dogs (16.7%) that did not express CD34 had cranial mediastinal lymphadenopathy. Five (41.7%) of 12 dogs with substage “b” expressed CD34, whereas none of the 11 dogs with substage “a” expressed CD34; four out of five dogs (80%) with CD34+ had fever, whereas only four of 18 dogs (22.2%) with CD34-had fever. The odds of cranial mediastinal lymphadenopathy were 20 times higher in CD34+ than those in CD34-dogs. CD34+ dogs were 2.6 times more likely to be evaluated as WHO clinical substage “b” than CD34-dogs, and their odds of having fever were 14 times higher.

A significant correlation was observed between the presence of MHCII expression and fever (*p* < 0.001), and the presence of chemotherapy adverse reactions (*p* = 0.022). Fever was present in seven of 10 MHCII+ dogs (70%), whereas none showed fever in the 13 MHCII-group. Chemotherapy adverse effects were present in seven of nine MHCII+ dogs (77.8%), whereas only two of 11 dogs with MHCII-showed chemotherapy adverse reactions. The odds of adverse reactions to chemotherapy were 16 times higher in the MHCII+ group than in the MHCII-group, and the odds of expressing MHCII were 5.3 times greater when fever was present than when fever was absent (Table 2).

**Table 2:** Association analysis between prognostic factors of lymphoma and aberrant immunophenotypes using Fisher’s exact test.

	Frequency (%)		Total
Cranial mediastinal lymphadenopathy	CD34+	CD34-	
Yes	4 (57.1%)	3 (42.9%)	7
No	1 (6.3%)	15 (93.8%)	16
Fisher’s exact test (*P*) [95% CI]	0.017 [1.613–247.981]		
WHO clinical substage	CD34+	CD34-	
a	0 (0%)	11 (100%)	11
b	5 (41.7%)	7 (58.3%)	12
Fisher’s exact test (*P*) [95% CI]	0.037 [1.441–4.589]		
Fever	CD34+	CD34-	
Yes	4 (50%)	4 (50%)	8
No	1 (6.7%)	14 (93.3%)	15
Fisher’s exact test (*P*) [95% CI]	0.033 [1.200–163.367]		
Chemotherapy adverse reactions	MHCII+	MHCII-	
Yes	7 (77.8%)	2 (22.2%)	9
No	2 (22.2%)	9 (81.8%)	11
Fisher’s exact test (*P*) [95% CI]	0.022 [1.754–141.404]		
Fever	MHCII+	MHCII-	
Yes	7 (100%)	0 (0%)	7
No	3 (18.8%)	13 (81.3%)	16
Fisher’s exact test (*P*) [95% CI]	<0.001 [1.923–14.790]		

### 3.3. Survival analysis

The overall median survival time was 365 days (range 1–1,138 days). Ten dogs died due to lymphoma, while two dogs died due to other reasons unrelated to the tumor. Twelve dogs survived the entire study duration, and follow-up was discontinued for three dogs.

No statistically significant differences in the survival period were observed for any of the phenotypic aberrancies. The survival time was significantly shorter in the substage “b” group (*p* = 0.006) and in the group with anemia and monocytosis among those with PNS (*p* = 0.028 and *p* = 0.024, respectively) (Supplementary Table S4).

## 4. Discussion

Companion animals serve as excellent models for human diseases, particularly spontaneously occurring cancers, which reflect similar pathobiologies and comorbidities. Dogs and humans share common cytogenetic and clinical features, pathology, tumor biology, tumor behavior, and genetic aberrations in the case of lymphoma (24, 25). In this study, flow cytometry was used to analyze immunophenotypes, an important prognostic factor in dogs diagnosed with lymphoma, and aberrancies were identified in 77% of the dogs (27/35 dogs). Dogs with high-grade lymphoma classified by the updated Kiel classification showed much more immunophenotypic aberrancies than ones with low-grade lymphoma. Furthermore, correlations between clinical, hematological, and serological findings were identified in dogs with aberrant phenotypes. As hypothesized, aberrancies associated with prognostic markers of lymphoma and PNS were identified, but no significant difference in survival time was observed according to the aberrant phenotype.

Previous studies have reported a slight difference in the incidence of aberrant phenotypes depending on the definition used. Specifically, Celant et al. found that 12% (310/2,612) of dogs had specific antigen aberrancies (20), while Wilkerson et al. reported an incidence of 22% (5). The incidence of loss of MHCII expression or low expression, which was the most frequently identified aberrancy in our study, has been reported to be approximately 14–72% in previous studies (2, 11). Additionally, the co-expression of CD3 and CD21 has been reported to range from as low as 0.7% (20), to as high as 31–50% (5, 19), while one study reported no cases of CD3/CD21 co-expression (26).

The MHCII proteins are specifically expressed on professional antigen-presenting cells such as B lymphocytes, monocytes, and dendritic cells (27). The MHCII gene expression signature suggests that antigen presentation to the immune system plays a significant role in therapeutic responses (27). The reduced MHCII expression may hinder sufficient tumor immunosurveillance, which could have contributed to the unfavorable outcome (27). In this study, the loss of MHCII expression was identified in the seven dogs with high-grade morphotypes (7/17, 41%). Although this study did not find a significant difference in survival time depending on MHCII expression, several previous human and dog studies have stablished its potential use as a prognostic factor.

CD3 is a complex molecule associated with the T-cell receptor and is expressed during maturation in early thymocytes (5). It is a representative marker expressed in T-cell lymphocytes and is present in all stages of T-cells from early precursor T-cells to mature T-cells that enter the circulation and lymph nodes. On the other hand, CD21 is a marker of mature B-cells, and when immature B-cells naïve to antigen exposure are released from the bone marrow, CD21 antigen is expressed on the surface (28).

In this study, the most frequently identified aberrancies in all lineages were double positive for CD3 and CD21. The co-expression of different lineage markers is a characteristic feature of tumors that cannot be identified in reactive lymph nodes (19). Four of the five dogs with low-grade lymphoma showed CD3+/CD21+, and three of them showed clear cell types and were diagnosed with TZL in histopathology. Eight dogs co-expressed CD21 among T cells, of which four dogs were presumed to have TZL and one dog had clinically aggressive multicentric T-cell lymphoma (no biopsy available). These were expected results since TZL is low grade indolent lymphoma and expresses CD21, as previously reported (29). However, in the case of the other dog, it was not clinically indolent. Six out of seventeen dogs with high-grade lymphoma showed CD3+/CD21+ expression, which is assumed to be aberrant expression.

CD21-positivity has already been described in canine T-cell neoplasms in the past (5, 19). Since it is a tumor cell, it is possible that the phenotype of the antigen expressed during the maturation stage of lymphocytes is altered. It is also possible that these two types of tumors occurred concurrently. One dog with CD3-expressing B-cell lymphoma was histopathologically diagnosed with DLBCL. However, in the clonality test, both T and B cells were confirmed to be clonal, suggesting that both tumor lineages occurred concurrently.

The loss of CD45, CD5, and CD4/CD8 double negativity was the second most common abnormality in T cells. CD45 is a transmembrane protein tyrosine phosphatase that serves as a common leukocyte marker due to its expression in all leukocytes irrespective of lineage (30). CD45 loss has also been regarded as a tumor hallmark in previous studies (19, 31). Among the histologic subtypes of lymphoma, CD45 is a characteristic finding of TZL, and as mentioned above, is clinically indolent. In our study, two dogs with CD45-were histopathologically diagnosed with TZL. One of the two dogs without histopathology had a tongue mass as the primary complaint and exhibited an immunophenotype of CD3+/CD4-/CD8+/CD21+/CD45-in the regional lymph nodes. Although the phenotype of the tongue mass was not analyzed, cytology confirmed that the main population was small lymphocytes (data not shown). The possibility of TZL originating from the tongue is also high in this dog, considering a previous case of TZL in the tongue (32). However, in another study, 5.3% of T-cell not otherwise specified dogs were identified as CD45- (20). Although this study did not find any aggressive lymphoma with CD45-, it is expected that the frequency of this aberrant type will increase as the population size increases.

T lymphocytes differentiate from double-negative (CD4-/CD8-) thymocytes to double-positive (CD4+/CD8+) cells before leaving the thymus and evolving into CD4+ and CD8+ cells (7). Since CD4 and CD8 are not expressed in mature T-cells, which are marked by CD3+, the phenotype observed in this study was considered aberrant. The CD4-/CD8-phenotype has been in various anatomical forms of T-cell lymphoma. For example, the phenotype of neoplastic lymphocytes in cutaneous epitheliotropic lymphoma was CD4-/CD8+ or CD4-/CD8- (33), and CD4-/CD8+ or CD4-/CD8-patterns were also identified in hepatosplenic and hepatocytotropic lymphoma (34). Regarding the survival period, a recent study found that dogs with a CD4-/CD8-/MHCII+ phenotype had a relatively long progression-free interval (2), while another study reported a more aggressive progression (35). Among lymphoma in dogs, lymphoblastic lymphoma, which has the most aggressive progression, has been shown to express CD4-/CD8-or CD4+/CD8+ (36). In this study, there was no significant difference in survival time, and no significant association with prognostic factors was confirmed. The four dogs with the CD4/CD8 double-negative phenotype did not show any common anatomical tumor regions.

In B-cells, MHCII-was identified in approximately 53% of cases, followed by CD3+/CD21+ in 41%, CD34+ in 24%, and CD79-in 24%. As previously mentioned, low MHCII expression is well known as one of the most reliable indicators of poor outcomes in human B-cell lymphoma (27, 37). Studies in dogs with B-cell lymphoma have also demonstrated an association between low MHCII expression and high mortality and relapse rates (11). There is evidence to suggest that MHCII expression is correlated with more robust immunosurveillance in B-cell lymphomas, as well as longer survival in T-cell lymphomas (12). However, in studies investigating MHCII expression in T-cell lymphoma, it remains unclear whether it can be used as a prognostic factor, as dogs with strong MHCII expression have been shown to have shorter survival times (2). Additionally, no difference in survival time was found depending on MHCII expression in a cohort of DLBCLs in a later study (38). Nevertheless, in this study, there was a strong correlation between MHCII expression and clinical signs and PNS caused by lymphoma, such as fever and cranial mediastinal lymphadenopathy, which suggests that MHCII expression may impact the severity or prognosis of the disease.

CD34 is a well-known hematopoietic precursor cell marker that has been clinically used to distinguish between acute lymphoblastic leukemia, chronic lymphocytic leukemia, and the leukemic stage (Stage V) of lymphoma (28). Although aberrant expression of CD34 has been observed in lymphoma without bone marrow involvement, its biological significance remains unclear (5, 6, 39). In case of precursor lymphoma, antigens expressed in lymphocytes at a relatively early stage of differentiation, including CD34, may also be identified. In this study, 19% (5/27) of dogs showed CD34 expression, and all showed high-grade morphotypes in the updated Kiel classification. Four of them were positive for CD21, a mature B-cell marker, suggesting aberrant expression in mature B-cell lymphoma. Among the T cells, one dog was identified as CD34+ and CD4-/CD8-, suggesting the possibility of precursor lymphoma. In previous studies, the expression rate of CD34 was 10–29%. When CD34 is co-expressed with CD21, a mature B-cell marker, as in this study, it is considered an aberrant phenotype (5, 11). Unfortunately, due to the lack of histopathology and a bone marrow analysis, the WHO classification and bone marrow infiltration was unknown. However, in the case of CD34+ B-cell lymphoma, considering various clinical and immunophenotypic characteristics, and tumor course, the possibility of acute leukemia or precursor lymphoma was estimated to be low. The response to chemotherapy of dogs with CD34+ B-cell lymphoma was poor, as it recurred during the induction (L-CHOP) protocol. Despite conducting a rescue protocol, the disease was considered progressive with a survival time of 99 days.

Next, the relationship between the phenotypic aberrations and clinical measurements was analyzed. The clinical significance of blood cell ratios as a biomarker has already been accepted for several diseases in humans, and investigations are being conducted on tumors of various origins in dogs. Inflammation plays a fundamental role in lymphomagenesis and tumor progression, and vice versa, and can lead to changes in peripheral blood leukocyte composition (neutrophil, monocyte, and lymphocyte, especially), depending on the severity and extent of inflammation (40, 41). PNR was confirmed as an independent prognostic factor in dogs diagnosed with DLBCL, with a cutoff value of 0.032; a higher value increases the risk of tumor progression before 180 days (42). Platelet–neutrophil interactions in malignant conditions are directly related to PNR, especially in humans and dogs with lymphomas presenting malignant hypercoagulability as a frequent PNS (42–45). In our study, PNR was significantly higher in the CD3+/CD21+ and CD45-group than in the group without this phenotype. Although there was no statistically significant difference in survival time among the PNR, CD45, and CD3+/CD21+ phenotypes, dogs with a low PNR generally tended to be ‘clinically ill’ upon admission. However, this was not related to tumor progression, and the relationship with phenotypic aberrancies could not be confirmed. Additionally, NLR was higher in the CD45+ group than in the CD45 + group. In human non-Hodgkin’s lymphoma, an NLR of 3.5 or higher was identified as a negative prognostic factor (46, 47). In this study, two out of four dogs with CD45-were diagnosed with TZL, and both had lymphocytosis at diagnosis, with neutrophils within the reference range. The difference in NLR is speculated to be largely due to the influence of lymphocytosis. However, the low NLR compared to lymphomas showing aggressive clinical features (e.g., DLBCL, peripheral T-cell lymphoma not otherwise specified) is probably due to the mild degree of tumor-induced inflammatory response (neutrophil change) and host immunity change (lymphocyte change) compared to aggressive lymphoma. This is presumed to be in line with the clinically indolent type and clinical signs similar to those of aggressive lymphomas, such as generalized lymphadenopathy and lymphocytosis, but different progression is thought to be related to changes in the blood cell ratio. The TZL population was too small to detect differences between TZL and other lymphomas or between TZL and healthy dogs.

Compared to the serum chemistry data, the concentration of ionized calcium was significantly higher in the CD3+/CD21+ group. However, their clinical relevance remains unknown. Among the dogs studied, two had hypercalcemia, which was evaluated as PNS due to parathyroid hormone related hormone, while one was found to have hypocalcemia. Several significant results were found in the Fisher’s exact test, most of which were related to PNS. Neoplastic fever is thought to be due to the innate immune response to a tumor antigen or the development of necrotic cells within the tumor, and is particularly common in hematopoietic cancers such as lymphoma (14). In this study, a significant difference in the presence or absence of fever was identified according to MHCII expression (*p* < 0.001). This is thought to be related to the function of MHCII and the mechanism of its upregulation in dogs with tumors. Cytokines such as TNF-α, IFN-γ, and IL-1 upregulate MHCII expression (48), and these cytokines also activate the arachidonic acid cascade to produce prostaglandin E_2_, which acts on the thermoregulatory center of the hypothalamus to regulate the development of fever (49). Although the direct causal relationship between MHCII expression and fever is unknown, further research is needed to determine the clinical relevance of MHCII as a negative prognostic factor and fever as a PNS. There was a correlation between MHCII expression and adverse reactions to chemotherapy as well. When MHCII was expressed, the risk of chemotherapy adverse reactions was higher than when it was not expressed. Most adverse reactions were myelosuppression, mainly neutropenia. Grade III or IV chemotherapy-induced neutropenia is known as a favorable prognostic factor of lymphoma in dogs (50, 51). Likewise, although the direct relationship between MHCII expression and chemotherapy adverse reactions could not be identified, it is presumed that there is an unknown mechanism between them.

In the case of CD34+ lymphoma, it can be presumed to be a precursor-derived or remaining aberrancy in the differentiation stage of tumor cells or leukemic involvement in high-grade lymphoma (52). WHO clinical substage “b” refers to a state with clinical signs such as lethargy, inappetence, weight loss, polyuria/polydipsia, or fever due to lymphoma, and it is a negative prognostic factor for lymphoma (1, 53). In this study, all 11 dogs in WHO substage “a” did not express CD34, while five out of 12 dogs (41.7%) in substage “b” expressed CD34. One of the five dogs expressing CD34 was of T-cell origin, and the other was of B-cell origin. There was no statistically significant difference in survival time, likely due to the small sample size, but four of the CD34+ dogs showed rapid tumor progression after or during the induction protocol (L-CHOP). Chemotherapy response in all four dogs was assessed as PD, and euthanasia was performed, or the dogs died due to tumor progression. When the correlation with cranial mediastinal masses, another negative prognostic factor, was analyzed, CD34 was expressed in four out of seven dogs (57.1%) with masses and only one (6.3%) out of 16 dogs without masses. Again, a causal relationship between these factors could not be confirmed, but it is presumed that there is a possible clinical association between CD34 expression, response to chemotherapy, and the progression-free interval.

When the Kaplan–Meier curve was used to analyze survival time based on the aberrant phenotype, no statistically significant difference in median survival time was observed for any phenotype. Previous studies have shown that the prognosis of T cells is poorer than that of B cells, but this study did not identify any differences in survival time according to immunophenotype. Although the dogs with high-grade lymphoma in the updated Kiel classification showed more immunopheonotypic aberrancies, it was not confirmed if it is related to the prognosis. There are prior studies that show that the prognosis is related to specific morphotypes with the updated Kiel classification (54). However, this study did not confirm the association with detailed morphotypes, immunophenotypic aberrations, and prognosis due to the small population. The survival period was significantly shorter in the PNS group, particularly in dogs with anemia and monocytosis. These results align with previously known negative prognostic factors for lymphoma. The small sample size and cases where tumor progression was not confirmed due to loss during follow-up may have contributed to these findings.

This study had some limitations associated with its retrospective nature. First, due to the small number of individuals, there were limitations in analyzing various aspects of phenotypic aberrations, which are known to have a particularly low frequency. In this study, two out of 19 dogs were identified as having non-B, non-T cell origin, and both were confirmed to be positive for the NK cell marker NKp46. Therefore, NK cell lymphoma could be diagnosed. However, it was difficult to compare the clinical characteristics because there were only two dogs. Second, there are still a few antibodies produced that target dogs for flow cytometry, and the majority of those used in this study are antibodies that cross-react with human, rat, or mouse cells. In this case, a false expression could be observed because of the risk of nonspecific binding. However, in this study, this was unlikely because the background cells, including neutrophils, monocytes, and reactive lymphocytes, did not show nonspecific binding to each antibody. Additionally, there are few antibodies available to further subdivide the phenotypes within the same lineage. By identifying phenotypic changes according to lymphocyte maturation stage and comparing them with clinical measurements, subtypes that are not yet widely known in veterinary medicine, such as precursor lymphoma, can be identified. Third, the WHO classification based on histopathology and bone marrow analysis remains a key tool for the diagnosis and classification of lymphoma and distinguishing it from bone marrow-originated diseases such as leukemia. In particular, a bone marrow analysis is necessary to determine whether the CD34 expression identified in this study is an aberrant immunophenotype, precursor lymphoma, or acute lymphoblastic leukemia, and peripheral blood tests are insufficient. Although it is not possible to evaluate the treatment response or prognosis using these methods alone, a clear evaluation is important as histological classification and grading is a strong prognostic factor. In clinical practice, surgical resection is not often attempted as diagnosis can typically be achieved to some extent with less invasive cytology and immunophenotyping. Future studies in populations with secured pathology results may enable accurate analysis between prognostic factors and clinical measurements. Such studies are expected to help our understanding of various subtypes of lymphoma in dogs by analyzing how the functions that are changed by aberrant antigen expression are clinically expressed at the gene expression level.

## Data availability statement

The original contributions presented in the study are included in the article/Supplementary material, further inquiries can be directed to the corresponding author.

## Ethics statement

The animal studies were approved by Gyengsang National University, IACUC no. GNU-230425-D0087. The studies were conducted in accordance with the local legislation and institutional requirements. Written informed consent was obtained from the owners for the participation of their animals in this study.

## Author contributions

HB: Conceptualization, Resources, Software, Writing – review & editing, Data curation, Formal analysis, Investigation, Methodology, Visualization, Writing – original draft. S-KK: Data curation, Formal analysis, Resources, Writing – review & editing, Funding acquisition, Validation. DY: Funding acquisition, Resources, Validation, Writing – review & editing, Conceptualization, Project administration, Software, Supervision.
