# Supplementary material for: Comparative analysis of the aberrant immunophenotype and clinical characteristics in dogs with lymphoma: a study of 27 cases
Source: Front Vet Sci. 2023 Oct 16;10:1254458. doi: 10.3389/fvets.2023.1254458 (PMC10613669; doi:10.3389/fvets.2023.1254458)
Supplement: Supplementary file 1 [file Data_Sheet_1.docx]

Supplementary Material

# Supplementary Table 1.

**Table S1.** Clinical characteristics and variables of 27 dogs diagnosed with aberrant lymphoma

| Characteristics | Case/total (N) | Percentage (%) |
| --- | --- | --- |
| Age, years; mean ± SD (range) | 9 ± 3.5 years (range, 3–15 years) | |
| ≤9 yrs | 12 | 44 |
| >9 yrs | 14 | 52 |
| Body weight, kg; mean ± SD (range) | 10 ± 10.48 kg (range, 2.2–41.6 kg) | |
| ≤10 kg | 19 | 70 |
| >10 kg | 4 | 15 |
| Sex | 26/27 |  |
| Female | 6 | 22 |
| Male | 0 | 0 |
| Spayed female | 11 | 41 |
| Neutered male | 9 | 33 |
| WHO clinical stage | 27/27 |  |
| I | 1 | 4 |
| III | 2 | 7 |
| IV | 13 | 48 |
| V | 11 | 41 |
| WHO clinical substage | 27/27 |  |
| a | 11 | 41 |
| b | 16 | 59 |
| Cell-type | 27/27 |  |
| Small | 5 | 19 |
| Intermediate | 7 | 26 |
| Large | 12 | 44 |
| Intermediate to large (heterogeneous) | 3 | 11 |
| Updated Kiel classification | 22/27 |  |
| Low grade | 5 | 19 |
| High grade | 17 | 63 |
| Anatomical form | 27/27 |  |
| Multicentric | 24 | 89 |
| Extranodal (alimentary, hepatic, tongue) | 3 | 11 |
| Immunophenotype | 27/27 |  |
| B-cell | 17 | 63 |
| T-cell | 8 | 30 |
| Non B- & non T-cell | 2 | 7 |
| Prior administration of prednisolone | 27/27 |  |
| Yes | 8 | 30 |
| No | 19 | 70 |

SD, standard deviation; WHO, World Health Organization

**Table S1.** (Continued) Clinical characteristics and variables of 27 dogs diagnosed with aberrant lymphoma

| Characteristics | Case/total (N) | Percentage (%) |
| --- | --- | --- |
| Chemotherapy | 21/27 |  |
| L-CHOP | 14 | 52 |
| CHOP | 1 | 4 |
| COP | 1 | 4 |
| Doxorubicin single | 1 | 4 |
| Prednisolone alone | 1 | 4 |
| Chlorambucil | 3 | 11 |
| Response of treatment  (Chemo-, supportive therapy) | 23/27 |  |
| CR | 5 | 19 |
| PR | 6 | 22 |
| SD | 4 | 15 |
| PD | 8 | 30 |
| Paraneoplastic syndrome |  |  |
| Fever (neoplastic) | 9 | 33 |
| Anemia | 18 | 67 |
| Hypercalcemia | 2 | 7 |
| Thrombocytopenia | 10 | 37 |
| Monocytosis | 15 | 56 |
| Neutrophilia | 11 | 41 |
| Overall median survival time | 365 days (1–1,138 days) | |
| Lymphoma-related death | 10 | 37 |
| Death unrelated to lymphoma | 2 | 7 |
| Survived to end of study | 12 | 44 |
| Loss of follow-up | 3 | 11 |
| Median survival time in progressive disease | 99±12 days | |

L-CHOP, L-asparaginase + vincristine + cyclophosphamide + doxorubicin + prednisolone; CHOP, vincristine + cyclophosphamide + doxorubicin + prednisolone; COP, vincristine + cyclophosphamide + prednisolone; CR, complete response; PR, partial response; SD, stable disease; PD, progressive disease

**Table S2.** Case summary according to the updated Kiel classification and immunophenotyping.

| Malignant lymphoma subtype | Number | Immunophenotype  (flow cytometry) | | |
| --- | --- | --- | --- | --- |
|  |  | B | T | Null |
| Low-grade | 5 |  |  |  |
| Clear cells | 3 |  | 3 |  |
| Variants form (centrocytic-like) | 1 | 1 |  |  |
| Prolymphocytic-like | 1 | 1 |  |  |
| High-grade | 17 |  |  |  |
| Centroblastic (polymorphic subtype) | 7 | 5 | 2 |  |
| Polymorphic lymphomas with a centroblastic component (predominantly small cells) | 1 |  |  | 1 |
| Pleomorphic lymphoma | 2 |  | 2 |  |
| Small-noncleaved-cell high-grade lymphoma (Burkitt’s lymphoma like) | 3 | 3 |  |  |
| Small-noncleaved-cell high-grade lymphoma (plasmacytoid) | 3 | 3 |  |  |
| Anaplstic lymphoma | 1 |  |  | 1 |

**Table S3.** Classification of cases according to lymphoma grading (low- and high-) by the updated Kiel classification and immunophenotypic aberrancies.

|  | Low-grade | High-grade |
| --- | --- | --- |
| CD3+/CD21+ | 4 | 6 |
| CD3-/CD21- | 0 | 2 |
| CD4+/CD8+ | 1 | 0 |
| CD4-/CD8- (except B) | 1 | 4 |
| CD5+ (except T) | 1 | 0 |
| CD5- (except B) | 0 | 4 |
| CD79a- (except T) | 1 | 3 |
| CD34+ | 0 | 5 |
| MHCII- | 3 | 7 |
| CD45- | 3 | 0 |

**Table S4**. Survival analysis through Kaplan–Meier curve and Log-rank test according to aberrant immunophenotypes and clinicopathological parameters.

| Variable | Number | Median (days) | *P* value |
| --- | --- | --- | --- |
| MHCII |  |  |  |
| Yes (≥60%) | 11 | 120 |  |
| No (<60%) | 13 | 123 | 0.203 |
| CD3+/CD21+ |  |  |  |
| No | 21 | 99 |  |
| Yes | 11 | 228 | 0.540 |
| CD34 |  |  |  |
| No (<60%) | 19 | 120 |  |
| Yes (≥60%) | 5 | 195 | 0.262 |
| CD45 |  |  |  |
| Yes (≥60%) | 24 | 100 |  |
| No (<60%) | 4 | 261 | 0.095 |
| Substage |  |  |  |
| a | 11 | - |  |
| b | 16 | 95 | 0.006** |
| Anemia |  |  |  |
| No (HCT ≥37.3%) | 9 | - |  |
| Yes (HCT <37.3%) | 18 | 70 | 0.028* |
| Monocytosis |  |  |  |
| No (≤1.48×10^9^/L) | 12 | - |  |
| Yes (>1.48×10^9^/L) | 15 | 50 | 0.024* |
| Neutrophilia |  |  |  |
| No (≤11.64×10^9^/L) | 16 | - |  |
| Yes (>11.64×10^9^/L) | 11 | 50 | 0.056 |

**P*<0.05, ***P*<0.01, ****P*<0.001

MHCII, major histocompatibility complex class II; HCT, hematocrit
